# An Overview of Chili Leaf Curl Disease: Molecular Mechanisms, Impact, Challenges, and Disease Management Strategies in Indian Subcontinent

**DOI:** 10.3389/fmicb.2022.899512

**Published:** 2022-06-29

**Authors:** Prashant Raghunath Shingote, Dhiraj Lalji Wasule, Vaishnavi Sanjay Parma, Somnath Kadappa Holkar, Suhas Gorakh Karkute, Narsing Devanna Parlawar, D. M. J. B. Senanayake

**Affiliations:** ^1^Department of Agricultural Biotechnology, Dr. Panjabrao Deshmukh Krishi Veedyapeeth, Akola, India; ^2^Department of Agricultural Biotechnology, Vasantrao Naik College of Agricultural Biotechnology, Yavatmal, India; ^3^Indian Council of Agricultural Research (ICAR)-National Research Centre for Grapes, Pune, India; ^4^Division of Vegetable Improvement, Indian Council of Agricultural Research (ICAR)-Indian Institute of Vegetable Research, Varanasi, India; ^5^Deparment of Agriculture, Rice Research and Development Institute, Bathalagoda, Sri Lanka

**Keywords:** *Chili leaf curl virus*, begomovirus, whitefly, chili, *Geminiviridae*, integrated disease management

## Abstract

Leaf curl disease in a chili plant is caused mainly by *Chili leaf curl virus* (ChiLCV) (Family: *Geminiviridae*, Genus: *Begomovirus*). ChiLCV shows a widespread occurrence in most of the chili (*Capsicum spp*.) growing regions. ChiLCV has a limited host range and infects tomatoes (*Solanum lycopersicum*), potatoes (*S. tuberosum*), and amaranth (*Amaranthus tricolor*). The virus genome is a monopartite circular single-stranded DNA molecule of 2.7 kb and associated with α and β-satellites of 1.3 and 1.4 kb, respectively. The virus genome is encapsulated in distinct twinned icosahedral particles of around 18–30 nm in size and transmitted by *Bemisia tabaci* (Family: *Aleyrodidae*, Order: *Hemiptera*). Recently, bipartite begomovirus has been found to be associated with leaf curl disease. The leaf curl disease has a widespread distribution in the major equatorial regions *viz*., Australia, Asia, Africa, Europe, and America. Besides the PCR, qPCR, and LAMP-based detection systems, recently, localized surface-plasmon-resonance (LPSR) based optical platform is used for ChiLCV detection in a 20–40 μl of sample volume using aluminum nanoparticles. Management of ChiLCV is more challenging due to the vector-borne nature of the virus, therefore integrated disease management strategies need to be followed to contain the spread and heavy crop loss. CRISPR/Cas-mediated virus resistance has gained importance in disease management of DNA and RNA viruses due to certain advantages over the conventional approaches. Therefore, CRISPR/Cas system-mediated resistance needs to be explored in chili against ChiLCV.

## Introduction

Chili (*Capsicum annuum* L.) is one of the major spice crops belonging to the *Solanaceae* family. Chili is a native of South America. Portuguese, during the fifteenth century, introduced chili to India, and since then, India is recognized as a secondary center for *Capsicum* species diversity. *C. annuum* is the most cultivated species; whereas *C. baccatum, C. chinense*, and *C. frutescens* are restricted to homestead gardening in India (Dhaliwal et al., 2014). The global production of green and red chilies for the year 2018-2019 has been recorded at 38.02 and 4.25 million tons, respectively. Globally, India is one of the main contributors to red chili production and its export. India shares around 42.81% of the total world area of chili cultivation (FAO, 2021; http://www.fao.org/faostat/). In India, during 2019–2020, the production of red chilies was around 1.7 million tons from a cultivated area of around 0.68 million hectares as per the second advance estimate (NHB, 2020).

Viruses are the major constraints in chili production worldwide because, at present, nearly 75 viruses are known to infect chili, of which 37 are the International Committee on Taxonomy of Viruses (ICTV) recognized species and six are tentative species (Kenyon et al., 2014; Thomas et al., 2021). In the recent past, chili leaf curl disease (ChiLCD) has emerged as a serious constraint to chili production in the Indian subcontinent (Kumar et al., 2011; Roy et al., 2019). Moreover, the re-emergence of new begomovirus strains due to recombination and mutations has endangered sustainable chili production globally (Juarez et al., 2019). ChiLCD is the most severe disease and is known to cause 100% losses to the marketable fruits (Kumar et al., 2011; Senanayake et al., 2012; Thakur et al., 2018). The severity of ChiLCD increases when it occurs in mixed infection with thrips or mites (Menike and De Costa, 2017; Thakur et al., 2018). In India, the first report of the occurrence of *Tomato leaf curl New Delhi virus* (ToLCNDV) was demonstrated by Khan et al. (2006). Recently, natural mixed infections of ChiLCV, *Cotton leaf curl Multan virus* (CLCuMuV) and *Tomato leaf curl Gujrat virus* (ToLCGV) were also reported to occur in chili in India (Mishra et al., 2020b).

ChiLCV belongs to the genus *Begomovirus* and the family *Geminiviridae*. The *Geminiviridae* family includes around 520 virus species, which are further divided into the 14 genera on the basis of genome organization, nucleotide sequence similarity, host range, and transmission (Thomas et al., 2021). The 14 genera are composed of various species *viz*, Becurtovirus (three species), *Begomovirus* (445 species), *Capulavirus* (four species), *Citlodavirus* (four species), *Curtovirus* (three species), *Eragrovirus* (one species), *Grablovirus* (three species), *Maldovirus* (three species), *Mastrevirus* (45 species), *Mulcrilevirus* (two species), *Opunvirus* (one species), *Topilevirus* (two species), *Topocuvirus* (one species), and *Turncurtovirus* (three species) as per the recent updates on recognized virus species by ICTV (Thomas et al., 2021). Among them, *Begomovirus* is one of the largest and most important genera which include several important species like ChiLCV. The genome of begomoviruses is composed of circular single-stranded DNA (ssDNA). The genome is encapsulated in a distinctive twinned-icosahedral particle of ~18–30 nm in size and is exclusively transmitted through whitefly (*Bemisia tabaci*; Order: *Hemiptera*, Family: *Aleyrodidae* (De Barro et al., 2011; Thakur et al., 2018).

Based on the genomic constitution, the begomoviruses are classified into three main types: (i) mono-partite begomoviruses having a single DNA genome that is analogous to the DNA-A genome of bipartite begomoviruses, (ii) monopartite begomoviruses with satellite ssDNA designated as β-satellite, and (iii) bipartite begomoviruses with two similar sizes of ssDNA segments designated as DNA-A and DNA-B (Seal et al., 2006; Ito et al., 2009; Roy et al., 2019). Begomoviruses are associated with various satellite molecules but the majority of ChiLCD has been reported to be caused by monopartite DNA-A viruses with β-satellite molecules (Hussain et al., 2004; George et al., 2014) whereas, limited bipartite type ChiLCV were also reported to cause ChiLCD (Kushwaha et al., 2015).

**Figure 1:**
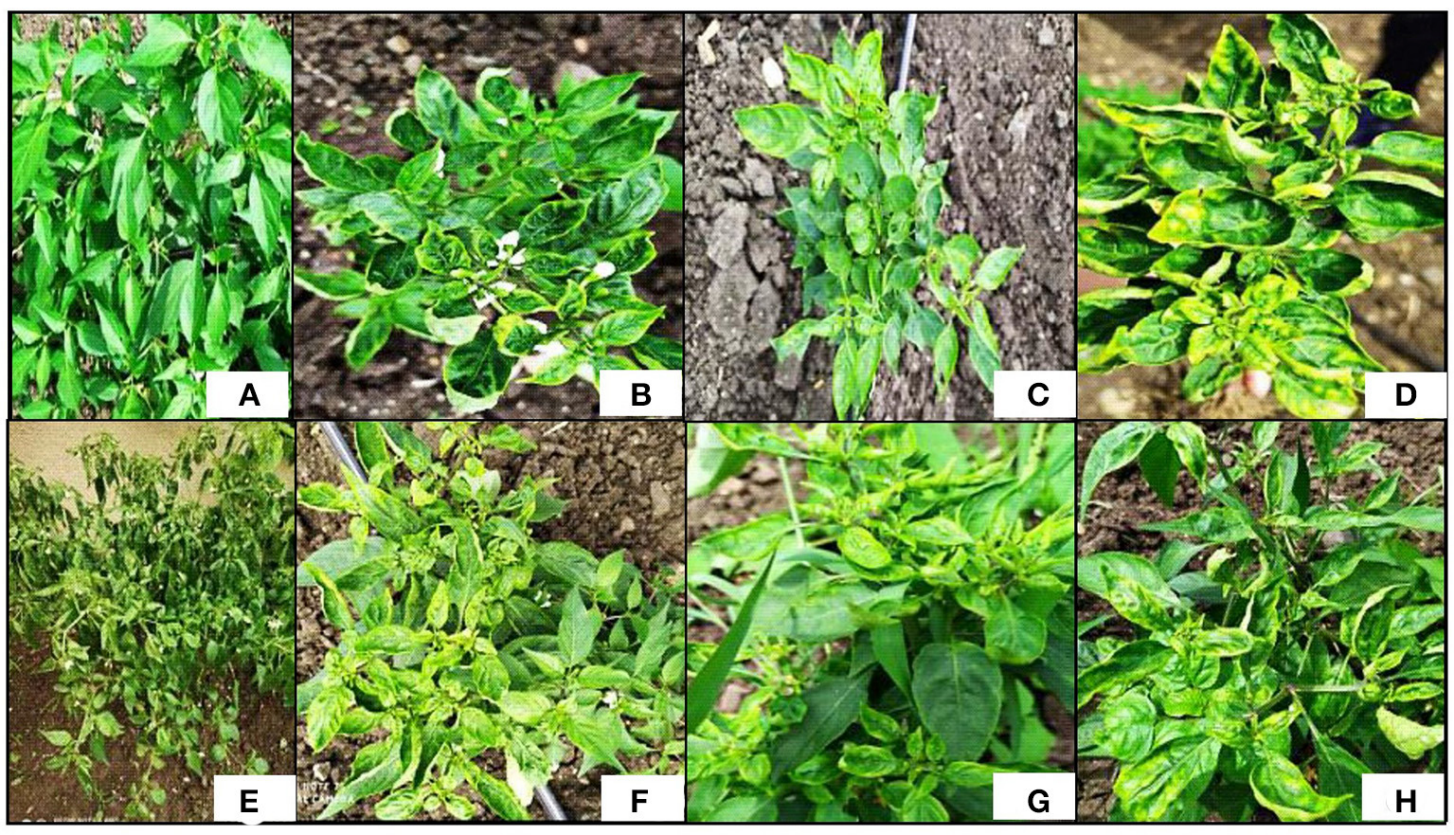
Symptoms of chili leaf curl disease (ChiLCD) in chili plants. **(A)** Healthy plant, **(B)** upward leaf curling, **(C)** vein banding, **(D)** upward leaf curling with yellow leaf margin, **(E)** bunching of leaves along with stunting of plants, **(F)** puckering and reduction in leaf, **(G)** shortening of internodes along with petioles, and **(H)** crinkling.

The symptoms of ChiLCV infection include upward curling in the leaves, crinkling appearance, puckering, leaf area reduction, blistering at interveinal areas, vein banding, shortening of petioles, and internodes, bunchy leaves, and severe stunting in plants (Figure 1; Kumar et al., 2014). If the disease persists till the late growth stages of the plants, flower buds show abscission and sterile anthers may set without or abnormal pollen grains, which ultimately produces poor fruit and low fruit setting, distorted or underdeveloped fruits (Kumar et al., 2015).

## Distribution of the Viruses Associated With ChiLCD

ChiLCD is reported and almost distributed in the equatorial regions of Australia, Asia, Africa, Europe, and America (Supplementary Figure 1, Table 1). ChiLCD was observed for the first time in India during the 1940's (Vasudeva, 1957). Subsequently, numerous other isolates of the ChiLCV have been identified throughout the world. There are several reports on other *Begomovirus* species causing ChiLCD in the Indian subcontinent and elsewhere (Thakur et al., 2018; Supplementary Table 1). At present 14 begomovirus species are reported to be associated with ChiLCD (Table 1). Among these, *Tomato leaf curl New Delhi virus* (ToLCNDV), *Chili leaf curl Palampur virus* (ChiLCPaV), ChiLCV, *Chili leaf curl Vellanad virus* (ChiLCVeV), *Chili leaf curl Salem virus* (ChiLCSV), *Chili leaf curl Gonda virus* (ChiLCGV), *Chili leaf curl Ahmedabad virus* (ChiLCAV), *Papaya leaf curl virus* (PaLCuV), *Chili leaf curl Bijnour virus* (ChiLCBV) from India, CLCuMuV and *Tomato leaf curl Joydebpur virus* (ToLCJoV) from Pakistan, *Pepper yellow leaf curl Indonesia virus* (PepLCIV) from Indonesia, and *Cabbage leaf curl virus* (CaLCuV) from Cuba, *Pepper leaf curl Bangladesh virus* (PepLCVBV) from Bangladesh have been reported (Shih et al., 2003; Hussain et al., 2004; Tsai et al., 2006; Kumar et al., 2011, 2014, 2015; George et al., 2014). The emergence of new biotypes of *B. tabaci* and the growing trend of the international market led to the spread of ChiLCD in different countries (Ramos et al., 2019). Moreover, expanding virus distribution by the introduction of novel crops in existing or new agricultural areas could resemble emergence of new begomovirus species or isolates driven by recombination and mutation (Varma and Malathi, 2003; Perefarres et al., 2012; Juarez et al., 2019).

**Table 1:** Distribution of begomoviruses associated with ChiLCD in Indian subcontinent.

**Virus species**	**Country**	**Host**	**References**
*Chili leaf curl Multan virus* (ChiLCMV)	Pakistan	*C. annuum*	Kumar et al., 2016
*Chili leaf curl Joydebpur virus* (ChiLCJV)	India	*C. annuum*	Shih et al., 2007
*Chili leaf curl Palampur virus* (ChiLCPaV)	India	*C. frutescens*	Kumar et al., 2011
*Chili leaf curl Vellanad virus* (ChiLCVeV)	India	*C. annuum*	Kumar et al., 2012
*Chili leaf curl Sri Lanka virus* (ChiLCSLV)	Sri Lanka	*C. annuum*	Fondong, 2013
*Chili leaf curl Salem virus* (ChiLCSV)	India	*Capsicum spp*.	Kumar et al., 2015
*Chili leaf curl Bijnour virus*	India	*Capsicum spp*.	Garcia-Neria and Rivera-Bustamante, 2011
*Chili leaf curl Ahmedabad virus* (ChiLCAV)	India	*C. annuum*	Ruhel and Chakraborty, 2019
*Chili leaf curl Gonda virus* (ChiLCGV)	India	*Capsicum spp*.	Khan and Khan, 2017
*Chili leaf curl virus* (ChiLCV)	India	*Amaranthus spp. Osteospermum fruticosum*	George et al., 2014; Shukla et al., 2016
*Tomato leaf curl New Delhi virus* (ToLCNDV)	India	*C. annuum*	Hussain et al., 2004
*Tomato leaf curl Joydebpur virus* (ToLCJoV)	India	*C. annuum*	Krishnan et al., 2019
*Pepper leaf curl Lahore* (PepLCLV)	Pakistan	*C. annuum*	Tahir et al., 2010

Around 39 species of whiteflies are known to transmit ChiLCD to the plants (Alemandri et al., 2015). *B. tabaci* infests around 600 plant species worldwide, and transmission of ChiLCV is caused by the viruliferous whiteflies by creating a feeding site in the phloem tissues of the host plant (Czosnek et al., 2017). Several viral isolates were found effective for transmission by whiteflies and all of which can produce typical symptoms of leaf curl disease in chili (Senanayake et al., 2012). The begomoviruses present in the vectors are transmitted in a circulative and persistent manner and infect dicotyledonous plant species (Thakur et al., 2018).

Surveys across some of the major chili-growing regions in India have been found that begomoviruses are complex in nature, with a diverse β satellite resulting in intra-specific recombination associated with ChiLCD (Kumar et al., 2015). Harmonious interface amongst chili infecting begomoviruses leads to the outbreak of the disease in resistant chili plants (Singh et al., 2016). The geographical distribution of ChiLCD is reported in parallel with the existence of whitefly in the world and found in equatorial regions of Australia, Asia, Africa, Europe, and America (Supplementary Figure 1). In India, ChiLCV associated with ChiLCD is distributed in different states *viz*., West Bengal, Bihar, Haryana, Delhi, Rajasthan, Uttar Pradesh, Himachal Pradesh, Goa, Gujarat, and Maharashtra (Kumar et al., 2015). Looking toward the devastating and widespread nature of the virus, it must be restricted to the infected regions only and should not spread to other parts of the globe. Worldwide efforts are being made toward understanding of nature of the virus and its economic importance and the development of resistant sources by means of conventional and non-conventional approaches.

## Diagnostics

Detection of the etiological agents is the foremost important aspect to devise efficient management strategies for any disease. Hence, an efficient, stable, robust, rapid, and reliable diagnostic procedure is a prerequisite step that facilitates the specific detection of plant pathogens. Earlier, plant disease recognition relied mainly on visual scouting of plant symptoms and their characteristics. However, this symptom-based observation was useful in some instances but it is ineffectual as a plethora of viruses show similar kinds of symptoms. With the recent advancements in many diagnostic techniques, sensitivity and effectiveness to detect the causal pathogen have been prioritized. Moreover, the serological and nucleic acid-based assays have gained momentum in diagnostics due to their rapidity, reliability, robustness, and accuracy.

The serological or immuno-assays such as enzyme-linked immunosorbent assay (ELISA), western blots, immunostrip assays, and dot-immuno-binding assays (DIBA) follow the principle of antigen-antibody interaction. Amidst all, an ELISA is the most prominent immunodiagnostic technique for the identification of viruses owing to its high throughput potential. Various formats of ELISA *viz*., triple antibody sandwich ELISA (TAS-ELISA), Direct antigen coated ELISA (DAC-ELISA), and double antibody sandwich ELISA (DAS-ELISA) have been routinely used with enhanced sensitivity. Likewise, radioactive probe-based dot-blot hybridization was used to determine the existence or identification of begomovirus in the chili samples (Senanayake et al., 2012). During the last two to three decades, tremendous developments in nucleic acid-based assays have happened, and therefore, virus diagnostics are being performed by these assays. Nevertheless, immuno-assays are proved to be the robust and reliable approach for routine virus indexing procedures in the laboratories engaged in plant-virus diagnostics and production of disease-free planting materials.

**Figure 2:**
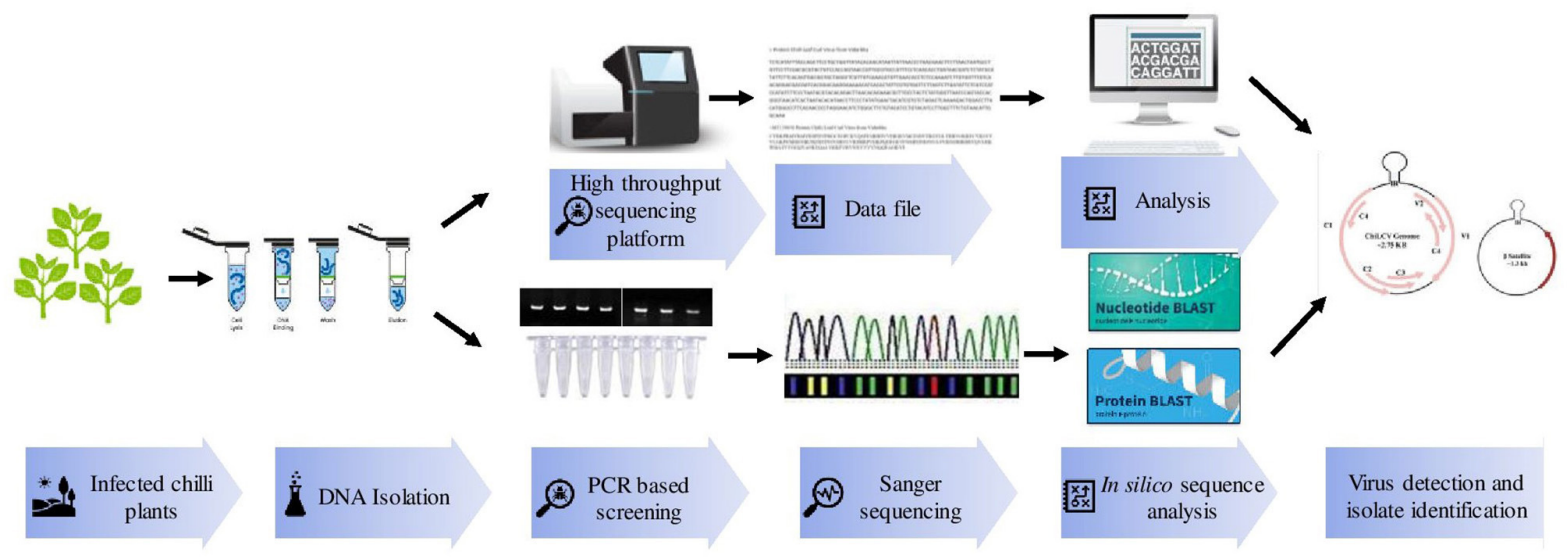
Various molecular techniques used for detection and identification of *Chili leaf curl virus* (ChiLCV) infecting chili.

Polymerase chain reaction (PCR) is a more efficient method than ELISA and hence provides a rapid, specific, and accurate detection of known viruses (Figure 2). ChiLCV has DNA genome thus, its detection is mainly confirmed through PCR using specific primers against coat protein (CP), intergenic regions (IR), and β-satellite regions (Briddon et al., 2003; Senanayake et al., 2012; Kumar et al., 2014). Further, in the recent past, there were numerous advancements in PCR assays for example quantitative real-time PCR (qPCR), multiplex PCR, and isothermal amplification have been practiced.

Isothermal amplification methods such as rolling circle amplification (RCA), recombinase polymerase amplification (RPA), and loop-mediated isothermal amplification (LAMP) have been introduced as an alternative to cost-deprived PCR assays. RCA using φ29 DNA polymerase mediated amplification was carried out in infected genomic DNA samples (Al-Shihi et al., 2014; Ranjan et al., 2016). In RCA, unidirectional DNA replication occurs which quickly synthesizes multiple copies of circular DNA molecules. These assays more importantly provide robust, rapid, and affordable detection. It is one of the cheaper, efficient, and simple methods which detect circular DNA viral genome when sequence information is unknown. Kumar et al. (2015) amplified components of the ChiLCV genome using RCA to decode the nature of begomovirus and β-satellite populations associated with ChiLCD across India. Besides, several researchers used RCA for the detection and characterization of ChiLCV (Venkataravanappa et al., 2016; Khan and Khan, 2017).

Similarly, qPCR has the ability to provide high sensitivity and specificity in the detection of viral diseases. The qPCR is widely used to detect viral DNA accumulation and study host-virus interaction genes, and viral expression (Kushwaha et al., 2015; Mangal et al., 2020). Multiplex PCR is another format of PCR, which allows the simultaneous detection of two or more virus species in a single reaction. Multiplex PCR assays have been optimized to amplify target DNAs at the same annealing temperature (Ranjan et al., 2016). However, multiplex PCR has not been yet utilized for the detection of ChiLCV. Advanced high throughput sequencing platforms become popular for the detection of viral loads. One such study reported the use of a Localized Surface-Plasmon-Resonance (LPSR) based optical platform for the detection of ChiLCV with a small volume (20–40 μL) of the sample. The LSPR absorbance signal could be monitored to detect the viral load and the sensitivity of LSPR assays by using aluminum nanoparticles for immobilization (Das et al., 2021).

## Genome Organization

**Figure 3:**
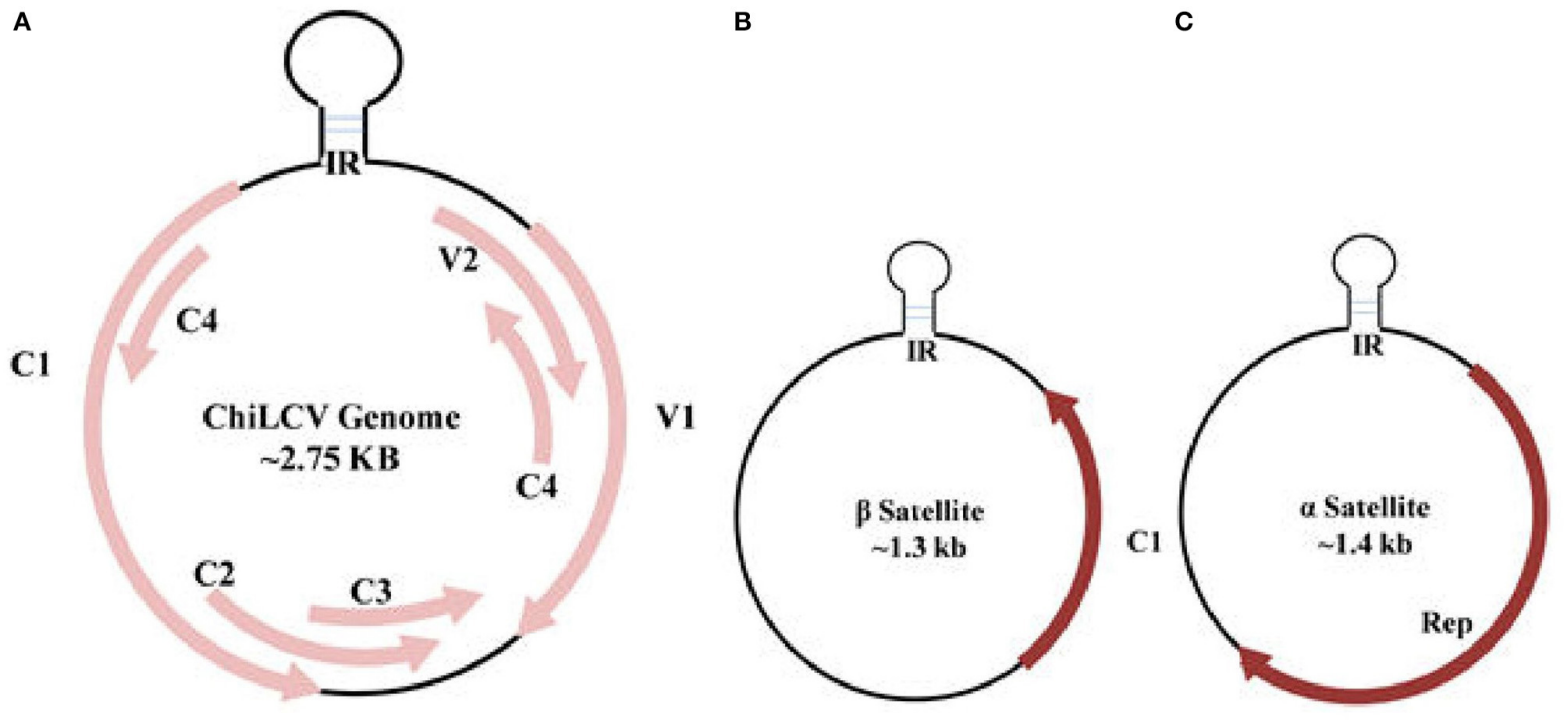
Genome organization of *Chili leaf curl virus* (ChiLCV) **(A)** and associated β and α satellites **(B,C)**. IR, intergenic region; Rep, replication initiator protein.

Among the 14 genera of the *Geminiviridae* family, *Begomovirus* is the largest plant virus genus comprising around 520 virus species. According to the recent ICTV classification, the genus recognition is based upon genome arrangement, trans-replication of the genetic constituent, and features of CP, vector, and host ranges (Thomas et al., 2021). *Begomovirus* genome consists of single-stranded circular DNA encompassing, the monopartite with a single component resembling DNA-A, or bipartite with two DNA components, DNA-A and DNA-B. Begomoviruses further distributed themselves in a “New World” as bipartite and predominantly as a monopartite in “Old World” viruses. Both the mono and bipartite genomes, each of around 2.75 kb in size, are independently enveloped in two incomplete icosahedral capsids geminate particles of about 20–30 nm (Hesketh et al., 2018). DNA-A encloses a gene required for encapsidation, replication, transmission, and gene suppression, whereas DNA-B harbors a movement protein that governs the export of viral DNA. DNA-B is not present in monopartite viruses, so the viral movement is mainly controlled by a CP and pre-CP of DNA-A (Poornima Priyadarshini et al., 2011). In association with the monopartite genome, a satellite molecule, and satellite-like components, namely α and β-satellite are often seen complementing DNA-A (Figure 3). An association with novel δ-satellite has also been reported in a few begomoviruses (Fiallo-Olive et al., 2016).

ChiLCV has a monopartite genome and the sequence analyses showed seven overlapping open reading frames (ORFs; Figure 3A). Out of seven ORFs, two are placed in the virion sense *viz*., V1 and V2; and five genes, AC1, AC2, AC3, AC4, and AC5, are located in the complementary sense strand (Figure 3A; Kumar et al., 2014). The genes V1 and V2 code for coat and pre-CP respectively, whilst AC1, AC2, and AC3 function as replication initiation protein (Rep), transcription activator protein (TrAP), and replication enhancer protein (REn) respectively, participating in gene expression and replication of viral genome in chili (Table 2). AC4 protein helps to determine symptom production whereas, the function of AC5 protein is still unclear. The transcriptional units of both the sense strand are separated by an intergenic region (Senanayake et al., 2013; Zerbini et al., 2017). Being of small genome size and obligate parasite most of the viral proteins are found to play multiple roles. The functions of ChiLCV proteins, the molecular weight of proteins, and the position of ORFs are listed in Table 2.

**Table 2:** The genome of *chili leaf curl virus* consisting of various open reading frames codes for different proteins having different functions in virus accumulation, movement, replication and post transcriptional gene silencing.

**Protein**	**ORF bases**	**Molecular weight (KDa)**	**Function**	**References**
Capsid protein (V1)	303–1,073	29.7	Cell-to-cell movement. Nuclear DNA Shuttling	Fondong, 2013; Wang et al., 2018
Pre-coat protein (V2)	197–490	11.7	Assist CP in cellular trafficking. Involved in TGS suppression.	Fondong, 2013; Kushwaha et al., 2017
Rep (C1)	1,516–2,640	42.7	Replication of ChiLCV genome. Modulate epigenetic modifications of viral DNA, Stimulate viral transcription	Fondong, 2013; Mei et al., 2018
TrAP (C2)	1,215–1,619	15.2	Transcriptional activator, suppressor of host RNAi silencing. Interfere ubiquitination pathway, and, inhibit plant JA defense.	Fondong, 2013; Li et al., 2018; Wang et al., 2018
REn (C3)	1,070–1,474	15.7	Enhances viral DNA accumulation/replication	Fondong, 2013; Wang et al., 2018
C4	2,160–2,450	10.7	Nucleocytoplasmic shuttle protein, aids nuclear export. Induce plant developmental abnormalities impairs plant triggered immunity/early defense response. Suppress gene silencing.	Fondong, 2013; Li et al., 2018
βC1	200–565	13.9	Enhance virus virulence. Inhibit TGS and PTGS of host defense. Repress JA responsive gene.	Fondong, 2013; Bhatt et al., 2016

### Intergenic Region

The IR region is the most prominent and highly conserved region of all the genera of *Geminiviridae* which is also designated as a common region (CR). In the ChiLCV genome, the IR consists of 279 nucleotides (nts) origin of replication with a 33-nts long potential stem-loop structure containing non-coding non-nucleotide sequence (TAATATTAC), which is required for cleavage and joining of the viral DNA during replication.

The replication-associated protein is encoded by the AC1 ORF which serves as the origin of replication and is expressed under the control of a bidirectional promoter (Bhatt et al., 2016). This Rep protein commences replication *via* the rolling-circle (RCR) mode of replication by introducing a nick at the phosphodiester bond of the seventh and eighth residues of non-nucleotide sequence. The cleaved 5' phosphate end of the sequence remains bound to Rep protein while the newly generated 3'-hydroxyl end starts the rolling-circle mode of replication (Ruhel and Chakraborty, 2019).

### α and β-Satellites

The β-satellite (~1.3 kb), is smaller in size than DNA-A and encodes a single conserved region of ~13–14 kDa protein known as βC1 in complementary orientation (Figure 3B). Apart from ORF, it also possesses a satellite conserved region (SCR) which is the adenine-rich region of ~150 nt (Briddon et al., 2003; Zhou, 2013). As a pathogenicity-determinant molecule, β-satellite *via* βC1 serves as a suppressor of RNA interference and also helps in determining the symptomatic phenotype through shifting the host defense system (Briddon et al., 2008; Patil and Fauquet, 2010). β-satellite mainly depends on the replication-associated protein of the helper viruses for replication and encapsidation. Another satellite, α-satellite, is about 1.4 kb codes for a Rep protein and is capable of self-replication that was earlier known to have an important role in the epidemiology of begomoviruses (Xie et al., 2010; Figure 3C). Unlike helper viruses, β-satellite do not own any conserved introns nor share sequence similarity with them or with α-satellites, except for the presence of stem-loop structure. Besides chili, variants of ChiLCV and associated satellite molecules tend to infect various new hosts such as *Amaranthus* species, potato (*Solanum tuberosum*), and tomato (*Solanum lycopersicum*; George et al., 2014; Venkataravanappa et al., 2016). Begomoviruses encode various proteins which are responsible for transcription activation, initiation of replication, encapsidation of genome, movement from cell to cell, and long-distance viral movement (Fondong, 2013).

### Genetic Diversity and Recombination

**Figure 4:**
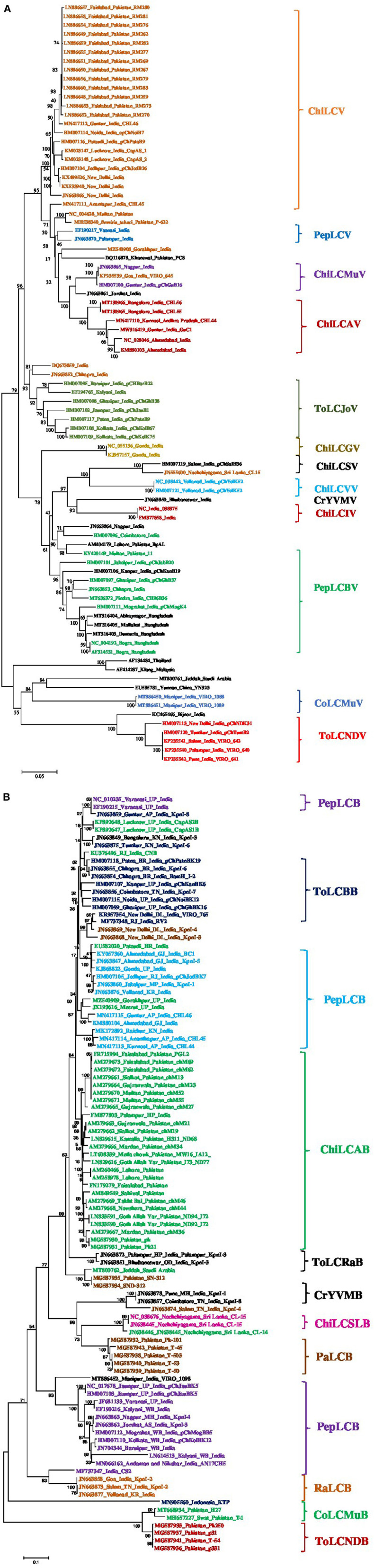
Phylogenetic dendrogram of begomoviruses **(A)** and betasatellites **(B)** associated with chili leaf curl disease (ChiLCD) in chili based on Neighbor-Joining method. The reference genome sequences were retrieved from NCBI GenBank for comparisons. Initially, the nucleotide sequences were aligned using Clustal W multiple alignment with bootstrap values of 1,000 replicates which are mentioned at the nodes. Major clades indicated in the specific color that belongs to the same or related species indicated at the right side of the each clade. The scale bar represents the rate of nucleotide substitutions per site.

The complete genome sequences of 84 isolates of begomoviruses associated with ChiLCD were retrieved from the NCBI GenBank and subjected to phylogenetic dendrogram construction. The phylogenetic tree revealed the 14 distinct clades (Figure 4A). The sequence identity matrix revealed the ChiLCAV shared 93–94% with ChiLCMuV therefore these two virus species are closely associated and reflected in one clade. ChiLCAV shared 93–100% sequence identity among them. ChiLCV shared 93% sequence identity with the ChiLCSV whereas, 91–94% with the PepLCV and 96–100% among themselves. Therefore, the close clustering of the PepLCV and ChiLCV was observed (Figure 4A). ToLCJoV shared 93% identity with the ChiLCV-Chhapra isolate. Whereas, PepLCV shared 90–93% of its identity with the other isolates of ChiLCV. PepLCV shared 91–93% with the ChiLCV and therefore clustered closely in the phylogenetic tree (Figure 4A). Similarly, the sequences of β-satellites were retrieved from the NCBI GenBank and constructed phylogenetic analyses and revealed the distinct 12 clades (Figure 4B).

Recently, Pandey et al. (2022) ascertained the genetic diversity of ChiLCD based on 121 reference genome sequences of DNA-A, DNA-B, and α and β-satellites. Moreover, transitional and transversional bias was recorded highest in C4 and REn genes. The 49 recombination breakpoints were estimated in C1, C2, C4, and V1 regions while a maximum of 22 breakpoints for Rep (AC1) and nine for βC1 were determined. A total of 2,505,419,807 and 1,288 mutations were detected for DNA-A and DNA-B, and α and β-satellites. A total of 987 mutations were also detected in the Rep gene which was the maximum among all ORFs. Based genetic mutations and recombination events evidenced that the ChiLCD rapidly adapted to new environments and led its evolution along with the satellite molecules that help in expanding the virus-host range and diversity in Indian subcontinents (Kumar et al., 2015; Pandey et al., 2022). Natural mixed infections of a bipartite and monopartite might have facilitated their emergence in new hosts, severity, and recombination.

## Epidemiology

In India, the first occurrence of ChiLCV in chili was reported in 2007 (Senanayake et al., 2007). Since then, several epidemics of ChiLCD in Central and Southern India the recent past has witnessed (Senanayake et al., 2007; Kumar et al., 2016; Chaubey and Mishra, 2017; Oraon and Tarafdar, 2018). ChiLCV infection is distributed in both the tropical and sub-tropical regions around the globe where different hosts are available (Senanayake et al., 2007; Supplementary Figure 1). The climatic conditions like a sudden rise in temperature, rainfall and relative humidity, intensification of cropping systems, and presence of alternate hosts enhance the whitefly population which leads to the rapid spread of begomoviruses and disease incidence (Kenyon et al., 2014). Warm weather conditions favor whitefly multiplication on host plants and there is a rapid increase in whitefly population even in the presence of natural enemies. Initially, under field conditions about 15–25% of chili plants have been observed with typical leaf curl symptoms and whitefly transmitted ChiLCV rapidly to reach about 50–100% infection in the field (Senanayake et al., 2007). The ChiLCD incidence is directly correlated with the abundance of the whitefly population. *B. tabaci* is polyphagous in nature, and it shows a high frequency of reproduction and quicker dispersal ability thus, facilitating its existence in large groups of populations with different agro-ecological zones. Even single whitefly has the ability to transmit the virus, whereas the presence of eight or more whiteflies on a plant may result in 100% chances of transmission (Senanayake et al., 2012). In an epidemic spread, several isolates of ChiLCV can be effectually transmitted by whiteflies, all of which can produce mild to severe characteristic symptoms in chili (Senanayake et al., 2012).

*B. tabaci* can complete around 12–15 overlapping generations in a year. The young and adult flies suck the sap and colonize on the ventral surface of the leaves. If activities like quick acquisition, fast transmission into the host, and faster lifecycles are maximized at a single geographical location then it may lead to the abrupt development of a new *Begomovirus* strain. *B. tabaci* transmits begomoviruses in a persistent manner which enables the spread of the virus through the juvenile, pupal, or adult stages of the whitefly. Adults of *B. tabaci* could not fly efficiently however, can be transported for quite a long distance by the wind. Begomoviruses have the ability to manipulate the behavior of their vectors this may improve their rate of transmission, consequently, these viruses affect the longevity and fertility of whitefly. However, the feeding habits and behavior of whiteflies can affect the population's genetics, behavior, and virus evolution (Holkar et al., 2017; Sangeeta and Tiwari, 2017). Recently, the various transmission parameters of ChiLCV were estimated for the development of the simulation of ChiLCD dynamics for devising management strategies. Subsequently, Roy et al. (2021) developed spatio-temporal distribution of whitefly population abundance in Indian conditions. The transmission deciding traits in tripartite interaction were estimated to simulate the ChiLCD dynamics by the population dynamics model framework. It has been evidenced that the ChiLCD epidemic is borne by a rigorous transformation frequency of a healthy host population into an infectious one (basic reproduction rate of whitefly: *R*_0_ = 13.54). Moreover, the least immigration frequency of vector was found to be essential in deciding the frequency of infectious host and viruliferous vector. In addition to this, migration of viruliferous vectors, virus acquisition, and transmission frequency were the important traits in deciding the epidemic of ChiLCD. Therefore, by keeping in view the effect of temperature (15–35°C) and transmission phenomenon in the build-up of virus-vector population, spatial and temporal pattern of disease risk has been predicted with ChiLCD distribution in India. This assessment of chili leaf curl disease dynamics would certainly be helpful to the growers in devising effective crop and disease management approaches (Roy et al., 2021).

## Transmission

For transmission of begomoviruses in tomatoes, whitefly requires a minimum of 90 min of acquisition and 120 min of inoculation feeding periods (Czosnek et al., 2002). *B. tabaci* are the lonely mediator to transmit begomoviruses through a circulative-persistent manner. Upon entry of the virus by ingestion it gets translocated via the digestive system to hemolymph, the salivary glands, and then expelled in the phloem of the plant. For the transmission of begomoviruses, the vector possesses many proteins which facilitate efficient viral transmission. During the infection process, the insect vector delivers viral particles presumably in the uncoated form to the plant and the viral genome is transported into the host cell nucleus. The viral genome encodes only a few proteins therefore, for their DNA replication they rely largely on the cellular DNA replication proteins. *Begomovirus* particles of 22 × 38 nm in size have two (geminate) incomplete T = 1 icosahedral capsid particles containing viral CP and each CP is bound by seven bases of viral ssDNA (Hesketh et al., 2018). As CP is the only structural protein in the geminiviral capsid, it is necessary for viral capsid assembly. In addition, CP also plays a crucial role in viral DNA transportation by interacting with cellular transporters as exemplified by mono-partite begomoviruses (Sharma and Ikegami, 2009). The CP of several monopartite begomoviruses possess a nuclear localization signal and a leucine-rich nuclear export signal and are thus localized to the nucleus (especially nucleolus), cytoplasm, and cell periphery (Unseld et al., 2001). A rich variety of interactions has evolved between viral proteins and host factors to develop the virus replicative cycle. In the first stage, once the viral genome is released from the virus capsid, it enters in to the cytoplasm of the host cell and subsequently enters the nucleus, where it undergoes rolling-circle and recombination-dependent replication process (Gutierrez, 1999). These newly replicated viral ssDNAs can be (1) converted into dsDNA which can act as a template for another round of replication or transcription, (2) wrapped by viral movement proteins for transportation from the infected cell to adjacent cells through plasmodesmata, or (3) encapsidated into infectious virions for long-distance virus movement (Gutierrez, 1999; Hanley-Bowdoin et al., 2013). The complex of viral DNA movement in begomoviruses has the ability to bind ssDNA or dsDNA and can transport the viral DNA to neighboring cells. Furthermore, begomoviruses possess a limited coding potential and thus rely heavily on host proteins to complete their infection cycle. Begomoviruses depend on host enzymes for their replication and transcription processes, coordinate with several cellular mechanisms to modulate cell division, cell cycle, and manipulate host components at different cellular levels (Hanley-Bowdoin et al., 2013). Transmission of a monopartite genome alone can lead to infection, but the presence of DNA A or DNA B helper genomes plays an important role in symptom development. *Begomovirus* proteins have a significant impact on a variety of host cell pathways which include changes in plasmodesmata structure and function, host cell defense mechanisms, and changes in gene expression in the infected cell (Peele et al., 2001).

## Integrated Disease Management Approaches

Viral diseases are controlled by various means like reduction in the rate of infection, rate of spreading of infection, and severity of infection which can be achieved through several cultural and mechanical practices (Figure 4). As pesticide residues are of great concern in bell pepper and chili, non-chemical pest control techniques such as cultural and mechanical activities are essential to prevent pests and ChiLCV (Ou et al., 2019).

Being large genera of the *Geminiviridae* family, begomoviruses alone, are accountable for the enormous devastation of many economically important field crops. The epidemiology caused by these viruses is a global concern, and its management is a prime necessity. Recently, Roy et al. (2021) estimated the tripartite interaction of transmission of ChiLCV in a population dynamics model framework to develop suitable management strategies. The sudden emergence of the whitefly as a virus vector is more complicated and perturb for researchers worldwide because of its prevalent nature, and unpredicted occurrence in the specific region on diverse hosts. Thus, these capabilities of *B. tabaci* render their management and control quite challenging. Management of ChiLCV is based on control of vector transmission, limiting the virus epidemic, and minimizing the severe impact of the disease on crop yield.

### Cultural Practices

The management of the whitefly-transmitted viruses must be initiated during seedling production stages and need to continue throughout the cropping cycle. Eventually, these can be achieved by implementing cultural practices as it covers a wide range of activities. The use of physical barriers is an extremely adopted practice, these include the use of insect-proof mesh housing, shielding the crop from colonization of whitefly and associated virus. Another approach is the use of UV absorbing sheets/polyethylene films which are generally used for the protection of greenhouse crops (Mutwiwa et al., 2005). Soil mulching is one of the well-practiced cultural practices, which involves the use of yellow mulch (polythene covering the soil) and reflective mulch (silver /aluminum coated plastics). Both these mulches generally cover the developing plant canopy and as a result, this impedes the ability of whiteflies to realize the crop and delay the onset of virus infestation. Mass trapping of adult whiteflies is one of the extensively used measures which involve the use of yellow sticky traps. Practicing intercropping system and trapping crops that attract whiteflies more than the host crop is also an important strategy to control whiteflies. The use intercropping system and trap crops will be beneficial for some of the selected crops and may fail for other crops due to the emergence of the new virus species and their spread in the main crops (Lapidot et al., 2014). Therefore, it is difficult to disseminate and adopt the intercropping system, which further needs proper extension and precise knowledge from the growers. The primary cultural practices are inefficient in reducing the virus spread, however, the infection and damage caused by the viruses can be delayed as it prevents the vector-host interaction. These tactics are not self-sufficient to manage the vector-virus transmission but still are helpful in the integrated control system (Kenyon et al., 2014).

### Chemical Control Methods

Control measures for the high infestation of *B. tabaci* mostly rely on the application of a wide variety of insecticides. This technique is highly reliable because of its efficiency and convenience (Horowitz et al., 2011). Conventionally practiced insecticides were belonging to the class of organochlorines, organophosphates, carbamates, and pyrethroids. Diafenthiuron aromatic ether is one of the most widely used insecticides (Thakur et al., 2018). However, the profuse and intensive application of insecticides (with similar repeated chemical compounds) leads to the development of a resistance mechanism in whiteflies against particular insecticide classes. Thus, this has exacerbated the chemical control measure tactics. Subsequently, these insecticides have been replaced with a novel group of insecticides that were more specific to target and effective at low concentrations. Neonicotinoids (imidacloprid and thiamethoxam), the Ketoenols (spiromesifen and spirotetramat), Diamides (flubendiamide, chlorantraniliprole, cyclaniliprole, tetraniliprole, and cyantraniliprole), and insect growth regulators such as buprofezin, pyriproxyfen were among the novel classes of insecticides. Moreover, some researchers have reported the resistance development in *B. tabaci* against these new classes of insecticides too (Horowitz et al., 2020). Alone and continuous usage of toxic and harmful pesticides has adversely disturbed the environment and mankind. The usage of these toxic pesticides can be reduced and the efficiency can be elevated by implementing the alternative integrated control strategies which involve the use of a combination of pesticides and other management tactics (Naranjo and Ellsworth, 2009). The efficiency can also be enhanced by the use of a mixture of insecticides however, these insecticides must be used in label recommendations. Besides these synthetic insecticides, the use of natural plant product extracts or biopesticides has been mentioned in some contexts. The biopesticides, neem seed kernel extract, and neem oil are effective in the reduction of disease incidence. *Sapindus trifoliatus*, and *Solanum trilobatum* seed extracts, *the* leaf extract of *Clerodendrum aculeatum* and bark extract of *Terminalia arjuna* have been found effective biocontrol agents (Pandey et al., 2010; Chaubey et al., 2017).

### Biological Control of Virus-Vectors

As aforesaid, the profuse and uncontrolled use of chemicals has resulted in insecticide resistance in the population of whitefly and also impacted the environment. Thereby, alternative practice such as biological means of control management has been put into practice to combat unwanted effects of insecticides/pesticides on crops, the environment, abiding pest resistance, and resurgence. The potential biological control agents constitute the use of natural enemies against *B. tabaci*, which primarily include predators, parasitoids, and entomopathogenic fungi. Some of the effective predators include *Amblyseius swirskii, Neoseiulus cucumeris, Amblyseius tamatavensis, Coccinella septempunctata, Chrysoperla carnea, Clitostethus arcuatus, Oriusspp, Chrysopa sp*., *Sinea confuse*, etc. (Soleymani et al., 2016; Cavalcante et al., 2017). The ladybird beetle, *Serangium japonicum* is also considered as an important predator of whiteflies (Tian et al., 2017). Parasitoids of *Encarisa* sp. and *Eretmocerus* sp. have been reported as efficient biocontrol agents in whitefly management. The biological control practice of using solitary natural enemies might be difficult in suppressing the whitefly population. But, the effectiveness of biological control agents can be elevated by using a combination of any two types of natural enemies. Ou et al. (2019) reported the use of insect parasitoids *Eretmocerus hayati* in combination with entomopathogenic fungi *Cordyceps javanica* which resulted in highly efficient against *B. tabaci*. In integrated pest management approaches, the efficiency of the biological agents might get reduced by the application of insecticides. However, a proper assessment of the lethal effect of the use of insecticides on the biocontrol agents must be optimized to increase its efficiency.

## Conventional Methods

Conventionally, ChiLCV management has mainly relied on the application of insecticides to control the vector whitefly. The use of pesticides is less effective, expensive for farmers, and causes hazards to the environment, consumers as well as farmers (Borah and Dasgupta, 2012). Furthermore, the presence of pesticide residues limits the export of chili. Besides, the uses of insecticides to control the vector whitefly are mostly unproductive as they are usually applied after the appearance of symptoms wherein the virus might have already been transmitted (Kenyon et al., 2014). In spite of the fact that there are several challenges in ChiLCV resistance breeding in chili, efforts on identification and introgression of resistant traits through resistance breeding is a very important option for reducing the input cost, to get the good returns to the chili growers worldwide and that would help to avoid virus-vector epidemics in future.

### Identification of Resistant Source

Resistance breeding is considered the most important approach for the development of ChiLCD resistance in chili genotypes. Resistance breeding is based on the recognition of robust resistant sources which are free from disease symptoms by screening under native field conditions followed by confirmation of resistance through artificial inoculation. Recently, in India, there are reports on the identification of some ChiLCV resistant genotypes/accessions from *C. annuum* that includes BS-35, EC-497636, DLS-Sel-10, GKC-29, PBC-142, PBC-143, PBC-144, PBC-149, PBC 495, VI012005, WBC-Sel-5, and Kalyanpur Chanchal (Kumar et al., 2006; Kenyon et al., 2014; Singh et al., 2016; Srivastava et al., 2017). Dhaliwal et al. (2015) from Punjab, India, reported a hybrid CH-27 resistant to ChiLCD. Further ChiLCV resistant *C. chinense* accessions BG-3821 and “Bhut Jolokia” were reported in Mexico and India, respectively (Anaya-Lopez et al., 2003; Adluri et al., 2017). Garcia-Neria and Rivera-Bustamante (2011) characterized a novel resistance trait in BG3821 (*C. chinense*) governed by two major genes for two begomoviruses. Further, functional characterization of the genes showed that germin-like proteins gene CchGLP is a vital factor for begomovirus resistance in BG-3821 accession from *C. chinense* (Mejia-Teniente et al., 2015). This area of resistance breeding is quickly enhancing the existing research information on chili and will undoubtedly be an important component in the progress of ChiLCD resistance development programs.

In one such experiment, Kumar et al. (2006) screened 307 genotypes under field and glasshouse conditions from five *Capsicum* sp. against ChiLCD and identified three genotypes *viz*., BS-35, EC-497636, and GKC-29 without symptoms. Same symptomless genotypes were further checked by grafting and alternate grafting with Pusa Jwala and the results confirmed the resistant reactions. Thus, these three genotypes showed characteristics of possible ChiLCD resistance donors. The GKC-29 and BS-35 are *C. frutescens* landraces from the North-East region of India, while EC-497636 was from Hungary. In another study, Rai et al. (2014) standardized an artificial microcage inoculation technique and elucidated the monogenic recessive nature of ChiLCD resistance. Adluri et al. (2017) also used Bhut Jolokia germplasm from North East India for screening of ChiLCV resistance. Bhutia et al. (2015) studied genetic control of ChiLCD severity traits in chili and found a cross of BCCHSel-4 × AC-575 promising for the level of field tolerance against ChiLCD. Srivastava et al. (2017) screened 65 different chili lines for resistance against begomoviruses causing ChiLCD under field conditions and found that DLS-Sel-10, PBC-142, and WBC-Sel-5 lines showed virus resistance. Recently, Jindal et al. (2018) evaluated the inheritance of ChiLCD resistance in chili where they identified S-343 line which may be used as a potential resistant donor for developing ChiLCD resistance cultivars. In DLS selection (DLSSel10/PhuleMukta/DLS-Sel.10) population, a single recessive gene was governing ChiLCD resistance in chili (Maurya et al., 2019). Further, resistance governed by a single dominant gene in S-343 has been documented by Thakur et al. (2019). It was evidenced that the genetics of resistance by crossing MS 341 (susceptible parent) with S-343 (resistant parent) wherein, after screening in F_2_ generation, it segregated into 3:1 plants whereas, susceptible parent backcross followed typical 1:1 test cross ratio suggesting the resistance is controlled by a single dominant gene. The reported ChiLCD resistant sources in chili are summarized in Supplementary Table 1.

The reported resistant sources failed to withstand the field conditions with an outbreak of disease due to a breakdown of defense barriers (Singh et al., 2016). They documented begomovirus-mediated reduced expression of host defense genes and outbreak of the disease in resistant chili plants. Recently, Thakur et al. (2020) mapped ChiLCV resistance gene using SSR markers. The study revealed that the two markers CA 516044 (6.8 cM) and PAU-LC-343-1 (8.9 cM) were associated with the ChiLCD resistant gene (located on chromosome number 6). Despite several reports, there are very less promising resistant donors that have been characterized in India. Characterization of such valuable resistant donors and associated markers will be highly desirable for resistance breeding against ChiLCD and its further introgression into other elite chili varieties. In spite of the number of identified resistance sources, breeding for resistance to ChiLCV has been limited due to the recessive nature of the resistance genes and the highly plastic genome of the begomoviruses. Furthermore, the reason for the inaccessibility of prominent resistant lines is the lack of a robust screening method for the identification of resistant lines and the evaluation of inheritance patterns. The present-day screening methods used are biolistic, grafting inoculation method, and whitefly inoculation techniques (Hernandez-Verdugo et al., 2001).

## Advanced Methods

**Figure 5:**
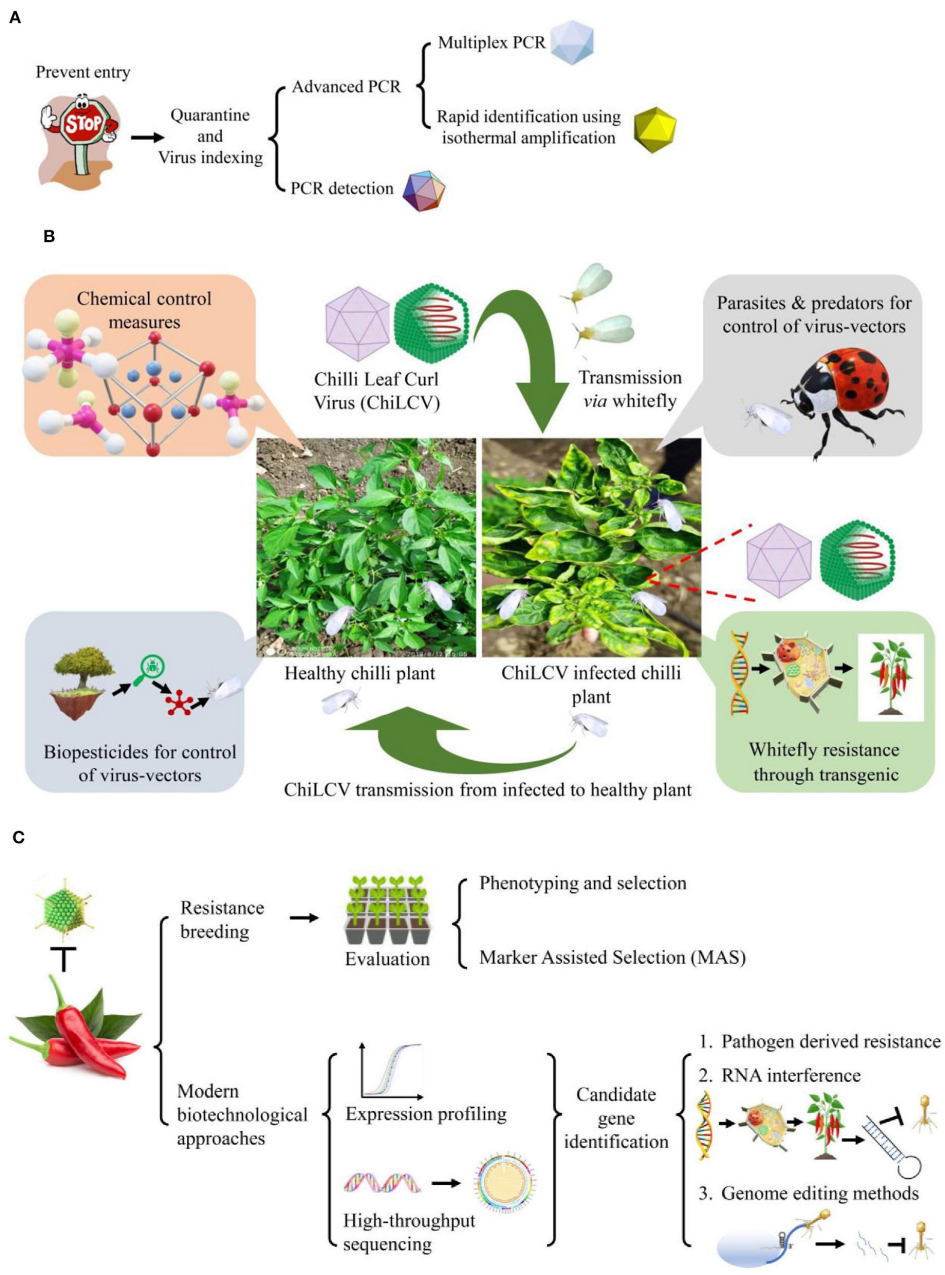
Conventional and non-conventional strategies for management of chili leaf curl disease (ChiLCD) in chili. **(A)** Strategies for *Chili leaf curl virus* (ChiLCV) management, **(B)** strategies for management of ChiLCD through virus-vector control, and **(C)** strategies for improvement of ChiLCD resistance in chili.

For management of ChiLCD in chili through non-conventional methods, there are two major approaches either direct or indirect strategies through control of virus-vector (Figure 5). The direct control approach includes pathogen-derived resistance (PDR), RNA interference (RNAi), and genome editing methods whereas the development of whitefly resistance in plants comes under the indirect approach (Figure 5).

### Pathogen Derived Resistance (PDR)

Classical methods of controlling virus infection have been proved to be unsuccessful against ChiLCD. The breeding approaches provided alternative methods to produce resistance sources however, it has its limitations lack resistant germplasm to *Begomovirus*es in chili. PDR is a more effective and fast approach for developing resistance against plant viruses in various crops (Powell et al., 1990). PDR strategy relies upon the post-transcriptional gene silencing (PTGS) mechanism for the successful utilization of virus-derived genes to get rid of viruses (Patil et al., 2011). Amongst PTGS, RNAi is one of the efficient technologies to induce resistance against viral pathogens. RNAi is a native defense antiviral system mediated through double-stranded RNA (dsRNA) which involves the degradation of sequence-specific viral RNA. Dicer-like proteins process the dsRNA into small 21–24 nts RNAs which interferes the viral RNA (Sharma et al., 2015).

In a study by Sharma et al. (2015), a demonstration of the ability of RNA silencing approaches to limit ChiLCV infection was attempted. Effectiveness was found through over-expression of AC1/AC2/βC1 specific dsRNA targeting various ChiLCV species. The PTGS strategy of RNAi through dsRNA results in the biogenesis of target-specific 21-24 nt small RNAs which governs begomovirus resistance in transgenic chili plants. Mishra et al. (2020a) identified chili miRNAs specific to essential genes of the ChiLCV through computational methods. They have predicted chili-encoded miRNAs targeting CP (V1) and Rep (C1) genes that could be used for silencing against ChiLCV infection. RNAi method will have to overcome some limitations like the ability to withstand natural field conditions and heavy loads of viruses, rapidly evolving ChiLCV variants that escaped sequence-specific recognition in RNAi mechanism, and also uncertainties about the release of genetically modified (GM) crops. Recently, Singh et al. (2022) a novel strategy of application of a cocktail of dsRNAs in *Nicotiana benthamiana* was attempted to prevent ChiLCV infection through RNAi. In this study, under *in vivo* conditions separate three dsRNA molecules were prepared using *E. coli* strain HT115 with the three suppressor genes, C2, V2, and C4 of ChiLCV. Further, an equal concentration of three dsRNAs (dsC2, dsV2, and dsC4) with 0.1% celite was used to prepare a cocktail of dsRNAs. A single spray of the cocktail of dsRNAs reduced ChiLCD incidence up to 66.7% in *N. benthamiana* plants for 2 weeks.

### Transgenic Approach

Biopesticides are potent microbial pesticides and biochemicals obtained from microbes and other natural resources. Advancement in plant genetic transformation technology has made it easy to overexpress the potent biomolecules into crop plants thereby conferring resistance against insect pests. Successful application of biopesticides depends on exact information of feeding habits and life cycle of the insect and the mechanism of action of the potent biopesticides. This facilitates determining the precise time and stage of biopesticide application for efficient results. To date, δ-endotoxin from *Bacillus thuringiensis* (Bt) is the most commonly used commercial biopesticide. The advantages of Bt biopesticides are they highly specific to target insects (*Lepidoptera, Coleoptera*, and *Diptera*). Nevertheless, the sap-sucking homopteran whiteflies remain insensitive to *Bt* toxin. Shukla et al. (2016) reported the *Tma12* gene from an edible fern (*Tectaria macrodonta*) has insecticidal activity against whitefly. *Tma12* overexpressing cotton transgenic lines were resistant to whitefly infestation and cotton leaf curl viral disease, with no detectable yield penalty. There is scope to identify such insecticidal proteins that are lethal to whiteflies. These biopesticide molecules need to be expressed in chili to overcome the whitefly-borne ChiLCV.

### CRISPR/Cas System

In the last decade, the CRISPR/Cas (clustered regularly interspaced short palindromic repeats and CRISPR-associated proteins) system has successfully emerged as one of the promising and precise genome-editing methods (Kale et al., 2021). The CRISPR/Cas system was reported for the first time in plants in the year 2013. Recently, gene-editing by using the CRISPR/Cas system has rapidly introduced genetic manipulations to achieve resistance against plant viral diseases (Ali et al., 2016; Tashkandi et al., 2018; Yin et al., 2019). Generally, two different mechanisms present in the CRISPR-Cas system governing antiviral activities *viz*., to recognize, interfere, and cleave the virus genome so that inhibition of multiplication of invasive viruses and to manage host susceptibility factors essential for virus replication and ultimately improve plant immunity and block the viral attack.

Severely affecting members of the family of *Geminiviridae* consist of around 485 ssDNA species. Multiple strategies have been initiated to destroy and get rid of the genomic DNA of begomoviruses through gene editing methods. Amongst the gene-editing methods, CRISPR/Cas system is more convenient for its simplicity in construction and design over the use of transcription activator-like effector nucleases (TALENs) and Zinc finger nucleases (ZFN). Therefore, this method has become more dominant and found promising for developing antiviral engineering in plants. In the transgenic model plant system, *N. benthamiana* CRISPR/Cas9 single guide RNA (sgRNAs) constructs specific to viral Rep, or IR region, showed effective DNA intrusion which thereby conferred resistance against CLCuMuV (Yin et al., 2019). One more study reported CP or Rep specific sgRNA mediated effective interference of tomato yellow leaf curl virus (TYLCV) genome in both the transgenic tomato and *N. benthamiana* lines (Tashkandi et al., 2018). Interestingly, Ali et al. (2016) reported that the stem-loop specific sgRNAs exhibited more efficient interference of various begomoviruses (Cotton leaf curl Kokhran virus, Merremia mosaic virus, and TYLCV) than the sgRNAs targeting CP and Rep regions of the viral genome (Ali et al., 2016).

CRISPR/Cas-mediated virus resistance in plants can also be established by targeting host factors. For plant virus protein translation, the recruitment of host-specific translation factors to facilitate their infection processes is required. Such host-specific translational factors are recognized and targeted as pro-viral factors. Further, in the recent past, numerous other pro-viral host factors, such as movement, replication, and metabolism-associated regions were characterized and used to limit plant virus diseases. eIF4E, eIF4G, and their isoforms are effective host translation initiation factors, which are exploited to achieve defense against various sub-species of viruses. Chandrasekaran et al. (2016) reported the eukaryotic initiation factor, eIF4E-knockout in cucumber through CRISPR/Cas9, which exhibited complete resistance to viruses involved in papaya ringspot, zucchini yellow mosaic, and cucumber vein yellowing diseases. Recent advancements in CRISPR/Cas9 technologies resulted in increasing reports of efficient plant DNA virus resistance. As an example, this CRISPR/Cas9 method targeting MP or CP region established resistance to TYLCV (Tashkandi et al., 2018). Roy et al. (2019) designed multiplexed sgRNA targeting ChiLCV genome and their approach was effective in removing the ChiLCV genome. Thus, CRISPR/Cas system becomes a potent tool for plant defenses against viruses and offers new avenues for genetic modification of chili against devastating ChiLCD. Although these advanced methods like RNAi and CRISPR/Cas systems holds the promise of achieving robust resistance, care has to be taken on various potential drawbacks associated with these methods.

Like host and virus, vectors can also be tapped for this novel technique, since, *Bemisia tabaci* is the only vector of begomoviruses and a serious pest of agricultural and horticultural crop plants. Recently, Heu et al. (2020) developed a CRISPR-Cas9 gene editing technique for silver leaf whitefly based on the vitellogenic adult females instead of embryos. The ovary-targeting peptide ligands were attached to Cas9 and inserted into adult females of *B. tabaci*. The offsprings were found to have a heritable edited genome. Thus, the development of a gene editing procedure for *B. tabaci* will helpful for the researchers to use the application of reverse genetics and will lead to the management of this serious insect vector.

## Conclusion

In recent years, there is a substantial increase in understanding of ChiLCD however, certain questions remain unanswered. The devastating and widespread nature of the ChiLCV highlights the urgency to recognize effective control measures for it. Whiteflies serve as a vector for numerous plant viruses and attempts to prevent whitefly population growth will be very beneficial in the field. The geographical distribution of ChiLCD is correlated with the incidence of whitefly in the world. Deciphering the effective environment-friendly biological control agents holds a promising key to control of the whitefly population. Further, whitefly-specific insecticidal proteins such as *Tma12* can be expressed in plants to restrict spread of vectors and ultimately the ChiLCV infection too. For resistance breeding, the reason for the inaccessibility of prominent resistant lines is the lack of a robust screening method for the identification of resistant lines and the evaluation of inheritance patterns. Advance molecular breeding holds the key to the identification of robust resistance sources and further introgression to elite lines. The advanced research needs to be concentrated on the identification of resistance sources using genomics-assisted breeding and developing robust resistance using breeding tools. Besides, RNAi against several transcripts of ChiLCV held the promise of being a fast and effective approach for achieving resistance against various plant viruses. Furthermore, genome editing based on the CRISPR-Cas system has offered a new capable technique for developing resistance against ChiLCV through alteration in host susceptibility factors. For achieving broad-range resistance against plant viruses through the CRISPR-Cas system various non-essential host susceptibility factors may prove to be more potent targets. Transgenic approaches looked to be very promising against ChiLCV, but the major hurdle for the release of transgenic varieties is uncertainties over government norms and public acceptance in India. In the detailed understanding of ChiLCV host–pathogen proteins integration, promotion, and the invention of novel technologies is the responsibility of the scientific community to get rid of ChiLCV.

## Author Contributions

PS, SH, and DS planned the review, drafted, and finalized. DW and VP prepared the tables, collected the literature, and drafting. SK prepared the figures and reference arrangements. All authors contributed to the article and approved the submitted version.

## Conflict of Interest

The authors declare that the research was conducted in the absence of any commercial or financial relationships that could be construed as a potential conflict of interest.

## Publisher's Note

All claims expressed in this article are solely those of the authors and do not necessarily represent those of their affiliated organizations, or those of the publisher, the editors and the reviewers. Any product that may be evaluated in this article, or claim that may be made by its manufacturer, is not guaranteed or endorsed by the publisher.

## References

[B1] Adluri P. K. Baldoldiya G. M. Nath P. D. (2017). Screening of Bhut Jolokia (*Capsicum chinense* Jacq.) germplasm of North East India against *Chili leaf curl virus*. Int. J. Pure Appl. Biosci. 5, 1189–1196. 10.18782/2320-7051.5624

[B2] Alemandri V. Vaghi-Medina C. G. Dumon A. D. Arguello-Caro E. B. Mattio M. F. Garcia Medina S.. (2015). Three members of the *Bemisia tabaci* (Hemiptera: *Aleyrodidae*) cryptic species complex occur sympatrically in Argentine horticultural crops. J. Econ. Entomol. 108, 405–413. 10.1093/jee/tov017 26470151

[B3] Ali Z. Ali S. Tashkandi M. Zaidi S. S. E. A. Mahfouz M. M. (2016). CRISPR/Cas9-mediated immunity to geminiviruses: differential interference and evasion. Sci. Rep. 6, 1–13. 10.1038/srep26912 27225592 PMC4881029

[B4] Al-Shihi A. A. Akhtar S. Khan A. J. (2014). Identification of *Chili leaf curl virus* causing leaf curl disease of Petunia in Oman. Plant Dis. 98, 572–572. 10.1094/PDIS-06-13-0678-PDN 30708693

[B5] Anaya-Lopez J. L. Torres-Pacheco I. Gonzalez-Chavira M. Garzon-Tiznado J. A. Pons-Hernandez J. L. Guevara-Gonzalez R. G.. (2003). Resistance to geminivirus mixed infections in Mexican wild peppers. Hortic. Sci. 38, 251–255. 10.21273/HORTSCI.38.2.251

[B6] Bhatt B. S. Chahwala F. D. Rathod S. Singh A. K. (2016). Identification and molecular characterization of a new recombinant Begomovirus and associated betasatellite DNA infecting Capsicum annuum in India. Arch. Virol. 161, 1389–1394. 10.1007/s00705-016-2769-z 26831933

[B7] Bhutia N. D. Seth T. Shende V. D. Dutta S. Chattopadhyay A. (2015). Estimation of heterosis, dominance effect and genetic control of fresh fruit yield, quality and leaf curl disease severity traits of chili pepper (*Capsicum annuum* L.). Sci. Hort. 182, 47–55. 10.1016/j.scienta.2014.11.017

[B8] Borah B. K. Dasgupta I. (2012). Begomovirus research in India: a critical appraisal and the way ahead. J. Biosci. 37, 791–806. 10.1007/s12038-012-9238-y 22922204

[B9] Briddon R. W. Brown J. K. Moriones E. Stanley J. Zerbini M. Zhou X.. (2008). Recommendations for the classification and nomenclature of the DNAβ satellites of begomoviruses. Arch. Virol. 153, 763–781. 10.1007/s00705-007-0013-6 18247103

[B10] Briddon R. W. Bull S. E. Amin I. Idris A. M. Mansoor S. Bedford I. D.. (2003). Diversity of DNA β, a satellite molecule associated with some monopartite begomoviruses. Virology 312, 106–121. 10.1016/S0042-6822(03)00200-9 12890625

[B11] Cavalcante A. C. C. Famah Sourassou N. de Moraes G. J. (2017). Potential predation of the exotic *Amblyseius swirskii* on Euseius concordis (Acari: Phytoseiidae), a predatory mite commonly found in Brazil. Biocontrol Sci. Technol. 27, 288–293. 10.1080/09583157.2016.1272096

[B12] Chandrasekaran J. Brumin M. Wolf D. Leibman D. Klap C. Pearlsman. (2016). Development of broad virus resistance in non-transgenic cucumber using CRISPR/Cas9 technology. Mol. Plant Pathol. 17, 1140–1153. 10.1111/mpp.12375 26808139 PMC6638350

[B13] Chaubey A. N. Mishra R. S. (2017). Survey of chilli leaf curl complex disease in eastern part of Uttar Pradesh. Biomed. J. Sci. Tech. Res. 1:589. 10.26717/BJSTR.2017.01.000589

[B14] Chaubey A. N. Mishra R. S. Singh V. (2017). Ecofriendly management of leaf curl disease of chili through botanical bio-pesticides. Plant Arch. 17, 285–291.

[B15] Czosnek H. Ghanim M. Ghanim M. (2002). The circulative pathway of begomoviruses in the whitefly vector *Bemisia tabaci*—insights from studies with *Tomato yellow leaf curl virus*. Ann. Appl. Biol. 140, 215–231. 10.1111/j.1744-7348.2002.tb00175.x

[B16] Czosnek H. Hariton-Shalev A. Sobol I. Gorovits R. Ghanim M. (2017). The incredible journey of begomoviruses in their whitefly vector. Viruses 9:273. 10.3390/v9100273 28946649 PMC5691625

[B17] Das S. Agarwal D. K. Mandal B. Rao V. R. Kundu T. (2021). Detection of the *Chili leaf curl virus* using an attenuated total reflection-mediated localized surface-plasmon-resonance-based optical platform. ACS Omega 6, 17413–17423. 10.1021/acsomega.1c01702 34278127 PMC8280655

[B18] De Barro P. J. Liu S. S. Boykin L. M. Dinsdale A. B. (2011). *Bemisia tabaci*? a statement of species status. Ann. Rev. Entomol. 56, 1–19. 10.1146/annurev-ento-112408-085504 20690829

[B19] Dhaliwal M. S. Jindal S. K. Cheema D. S. (2015). CH-27: a multiple disease resistant chili hybrid. Agricul. Res. J. 52, 127–129. 10.5958/2395-146X.2015.00078.2

[B20] Dhaliwal M. S. Yadav A. Jindal S. K. (2014). Molecular characterization and diversity analysis in chili pepper using simple sequence repeats (SSR) markers. Afr. J. Biotechnol. 13:695. 10.5897/AJB2014.13695

[B21] FAO (2021). World Food and Agriculture – Statistical Yearbook. Rome: FAO.

[B22] Fiallo-Olive E. Tovar R. Navas-Castillo J. (2016). Deciphering the biology of deltasatellites from the New World: maintenance by New World Begomoviruses and whitefly transmission. New Phytol. 212, 680–692. 10.1111/nph.14071 27400152

[B23] Fondong V. N. (2013). Geminivirus protein structure and function. Mol. Plant Pathol. 14, 635–649. 10.1111/mpp.12032 23615043 PMC6638828

[B24] Garcia-Neria M. A. Rivera-Bustamante R. F. (2011). Characterization of geminivirus resistance in an accession of Capsicum chinense Jacq. Mol. Plant Microbe Interact. 24, 172–182. 10.1094/MPMI-06-10-0126 20923365

[B25] George B. Kumar R. V. Chakraborty S. (2014). Molecular characterization of *Chili leaf curl virus* and satellite molecules associated with leaf curl disease of Amaranthus spp. Virus Genes 48, 397–401. 10.1007/s11262-013-1027-7 24368759

[B26] Gutierrez C. (1999). Geminivirus DNA replication. Cell. Mol. Life Sci. 56, 313–329. 10.1007/s000180050433 11212359 PMC11146802

[B27] Hanley-Bowdoin L. Bejarano E. R. Robertson D. Mansoor S. (2013). Geminiviruses: masters at redirecting and reprogramming plant processes. Nat. Rev. Microbiol. 11, 777–788. 10.1038/nrmicro3117 24100361

[B28] Hernandez-Verdugo S. Guevara-Gonzalez R. G. Rivera-Bustamante R. F. Oyama K. (2001). Screening wild plants of *Capsicum annuum* for resistance to pepper huasteco virus (PHV): Presence of viral DNA and differentiation among populations. Euphytica 122, 31–36. 10.1023/A:1012624830340

[B29] Hesketh E. L. Saunders K. Fisher C. Potze J. Stanley J. Lomonossoff G. P.. (2018). The 3.3 Å structure of a plant geminivirus using cryo-EM. Nat. Commun. 9, 1–10. 10.1038/s41467-018-04793-6 29915210 PMC6006435

[B30] Heu C. C. Francine M. M. Junbo L. Jason L. R. (2020). CRISPR-Cas9-based genome editing in the silverleaf whitefly (*Bemisia tabaci*). CRISPR J. 3, 89–96. 10.1089/crispr.2019.0067 32315225 PMC7194319

[B31] Holkar S. K. Kaushal P. Kumar S. (2017). “Host preference by evolving insect vectors in relation to infection of plant viruses,” in The Phytopathogen: Evolution and Adaptation, eds A. Ghatak and M. Ansar (Boca Raton, FL: CRC Press), 259–304. 10.1201/9781315366135-10

[B32] Horowitz A. R. Antignus Y. Gerling D. (2011). “Management of *Bemisia tabaci* whiteflies,” in The Whitefly, Bemisia tabaci (Homoptera: Aleyrodidae) Interaction With Geminivirus-Infected Host Plants, eds M. Winston and O. Thompson (Dordrecht: Springer), 293–322. 10.1007/978-94-007-1524-0_11

[B33] Horowitz A. R. Ghanim M. Roditakis E. Nauen R. Ishaaya I. (2020). Insecticide resistance and its management in *Bemisia tabaci* species. J. Pest Sci. 93, 893–910. 10.1007/s10340-020-01210-0

[B34] Hussain M. Mansoor S. Iram S. Zafar Y. Briddon R. W. (2004). First report of tomato leaf curl New Delhi virus affecting chili pepper in Pakistan. Plant Pathol. 53:1073. 10.1111/j.1365-3059.2004.01073.x

[B35] Ito T. Kimbara J. Sharma P. Ikegami M. (2009). Interaction of tomato yellow leaf curl virus with diverse betasatellites enhances symptom severity. Arch.Virol. 154, 1233–1239. 10.1007/s00705-009-0431-8 19575277

[B36] Jindal S. K. Dhaliwal M. S. Sharma A. Thakur H. (2018). Inheritance studies for resistance to leaf curl virus disease in chili (*Capsicum annuum* L.). Agric. Res. J. 55, 757–760. 10.5958/2395-146X.2018.00139.4

[B37] Juarez M. Rabadan M. P. Martinez L. D. Tayahi M. Grande-Perez A. Gomez P. (2019). Natural hosts and genetic diversity of the emerging tomato leaf curl New Delhi virus in Spain. Front. Microbiol. 10:140. 10.3389/fmicb.2019.00140 30842757 PMC6391364

[B38] Kale R. R. Shingote P. R. Wasule D. L. Mirajkar S. J. Rathod D. R. Moharil M. P. (2021). “Historical developments of genome editing in plants,” in Genome Editing in Plants, eds O. P. Gupta and S. G. Karkute (Boca Raton, FL: CRC Press), 1–11. 10.1201/9780367815370-1

[B39] Kenyon L. Kumar S. Tsai W. S. Hughes J. D. A. (2014). Virus diseases of peppers (*Capsicum spp*.) and their control. Adv. Virus Res. 90, 297–354. 10.1016/B978-0-12-801246-8.00006-8 25410105

[B40] Khan M. S. Raj S. K. Singh R. (2006). First report of Tomato leaf curl New Delhi virus infecting chilli in India. Plant Pathol. 55:289. 10.1111/j.1365-3059.2006.01324.x23637491

[B41] Khan Z. A. Khan J. A. (2017). Characterization of a new Begomovirus and betasatellite associated with chili leaf curl disease in India. Arch. Virol. 162, 561–565. 10.1007/s00705-016-3096-0 27738844

[B42] Krishnan N. Kumari S. Krishnan S. Dubey V. Singh A. K. Kumar R. (2019). First report of tomato leaf curl joydebpur virus infecting chilli (*Capsicum annuum*) in Andaman and Nicobar Islands. Plant Dis. 103, 2974–2974. 10.1094/PDIS-03-19-0451-PDN

[B43] Kumar J. Kumar J. Singh S. P. Tuli R. (2014). βC1 is a pathogenicity determinant: not only for begomoviruses but also for a mastrevirus. Arch Virol. 159, 3071–3076. 10.1007/s00705-014-2149-5 25000899

[B44] Kumar R. Kumar V. Kadiri S. Palicherla S. R. (2016). Epidemiology and diagnosis of chilli leaf curl virus in central India, a major chilli growing region. Indian Phytopathol. 69, 61–64.

[B45] Kumar R. V. Singh A. K. Chakraborty S. (2012). A new monopartite *Begomovirus* species, *Chili leaf curl Vellanad virus*, and associated betasatellites infecting chili in the Vellanad region of Kerala, India. New Dis. Rep. 25:20. 10.5197/j.2044-0588.2012.025.020

[B46] Kumar R. V. Singh A. K. Singh A. K. Yadav T. Basu S. Kushwaha N.. (2015). Complexity of Begomovirus and betasatellite populations associated with chili leaf curl disease in India. J. Gen. Virol. 96, 3143–3158. 10.1099/jgv.0.000254 26251220

[B47] Kumar S. Kumar S. Singh M. Singh A. K. Rai M. (2006). Identification of host plant resistance to *Pepper leaf curl virus* in chili (*Capsicum* species). Sci. Hortic. 110, 359–361. 10.1016/j.scienta.2006.07.030

[B48] Kumar Y. Hallan V. Zaidi A. A. (2011). *Chili leaf curl Palampur virus* is a distinct *Begomovirus* species associated with a betasatellite. Plant Pathol. 60, 1040–1047. 10.1111/j.1365-3059.2011.02475.x

[B49] Kushwaha N. Sahu P. P. Prasad M. Chakraborty S. (2015). *Chili leaf curl virus* infection highlights the differential expression of genes involved in protein homeostasis and defense in resistant chili plants. Appl. Microbiol. Biotechnol. 99, 4757–4770. 10.1007/s00253-015-6415-6 25693670

[B50] Kushwaha N. K. Bhardwaj M. Chakraborty S. (2017). The replication initiator protein of a geminivirus interacts with host monoubiquitination machinery and stimulates transcription of the viral genome. PLoS Pathogens 13:e1006587. 10.1371/journal.ppat.1006587 28859169 PMC5597257

[B51] Lapidot M. Legg J. P. Wintermantel W. M. Polston J. E. (2014). Management of whitefly-transmitted viruses in open-field production systems. Adv.Virus Res. 90, 147–206. 10.1016/B978-0-12-801246-8.00003-2 25410102

[B52] Li F. Yang X. Bisaro D. M. Zhou X. (2018). The βC1 protein of geminivirus–betasatellite complexes: a target and repressor of host defenses. Mol. Plant Pathol. 11, 1424–1426. 10.1016/j.molp.2018.10.007 30404041

[B53] Mangal M. Srivastava A. Mirajkar S. J. Singh K. Solanki V. Mandal B.. (2020). Differential expression profiling of defense related genes for Leaf Curl Virus (ChiLCV) in resistant and susceptible genotypes of Chili. Indian J. Genet. 80, 308–317. 10.31742/IJGPB.80.3.10

[B54] Maurya P. K. Srivastava A. Mangal M. Talukdar A. Mondal B. Solanki V.. (2019). Genetic analysis for resistance to leaf curl disease in Chili Peppers (*Capsicum annuum* L.) under specific situations. Indian J. Genet. 79, 741–748. 10.31742/IJGPB.79.4.13

[B55] Mei Y. Wang Y. Hu T. Yang X. Lozano-Duran R. Sunter G.. (2018). Nucleocytoplasmic shuttling of geminivirus C4 protein mediated by phosphorylation and myristoylation is critical for viral pathogenicity. Mol. Plant Pathol. 11, 1466–1481. 10.1016/j.molp.2018.10.004 30523782

[B56] Mejia-Teniente L. Joaquin-Ramos A. D. J. Torres-Pacheco I. Rivera-Bustamante R. F. Guevara-Olvera L. Rico-Garcia E.. (2015). Silencing of a germin-like protein gene (CchGLP) in geminivirus-resistant pepper (*Capsicum chinense* Jacq.) BG-3821 increases susceptibility to single and mixed infections by geminiviruses PHYVV and PepGMV. Viruses 7, 6141–6151. 10.3390/v7122930 26610554 PMC4690854

[B57] Menike G. D. N. De Costa D. M. (2017). Variation of field symptoms and molecular diversity of the virus isolates associated with chili leaf curl complex in different agroecological regions of Sri Lanka. Trop. Agric. Res. 28, 144–161. 10.4038/tar.v28i2.8192

[B58] Mishra M. Kumar R. Gaur R. K. Verma R. K. (2020a). *In silico* analysis of chili encoded miRNAs targeting chili leaf curl Begomovirus and its associated satellite. J. Appl. Biol. Biotechnol. 8, 1–5. 10.7324/JABB.2020.80101

[B59] Mishra M. Verma R. K. Marwal A. Sharma P. Gaur R. K. (2020b). Biology and interaction of the natural occurrence of distinct monopartite begomoviruses associated with satellites in *Capsicum annuum* from India. Front. Microbiol. 11:2366. 10.3389/fmicb.2020.512957 33117300 PMC7575687

[B60] Mutwiwa U. N. Borgemeister C. Von elsner B. Tantau H. J. (2005). Effects of UV-absorbing plastic films on greenhouse whitefly (Homoptera: *Aleyrodidae*). J. Econ. Entomol. 98, 1221–1228. 10.1603/0022-0493-98.4.1221 16156574

[B61] Naranjo S. E. Ellsworth P. C. (2009). The contribution of conservation biological control to integrated control of *Bemisia tabaci* in cotton. Biol. Control 51, 458–470. 10.1016/j.biocontrol.2009.08.006

[B62] NHB (2020). Horticulture Crops for 2019–20 (Second Advance Estimates), Area, and Production of Horticulture Crops: All India. New Delhi: Ministry of Agriculture and Farmers Welfare, Govt. of India, 3.

[B63] Oraon U. B. Tarafdar J. (2018). Occurrence and distribution of chilli leaf curl complex disease in West Bengal. Biomed. J. Sci. Tech. Res. 3, 3515–3519. 10.26717/BJSTR.2018.3.000948

[B64] Ou D. Ren L. M. Liu Y. Ali S. Wang X. M. Ahmed M. Z.. (2019). Compatibility and efficacy of the parasitoid *Eretmocerus hayati* and the entomopathogenic fungus *Cordyceps javanica* for biological control of whitefly *Bemisia tabaci*. Insects 10:425. 10.3390/insects10120425 31775366 PMC6956003

[B65] Pandey S. K. Mathur A. C. Manisha S. (2010). Management of leaf curl disease of chilli (*Capsicum annuum* L.). Intl. J. Virol. 6, 246–250. 10.3923/ijv.2010.246.250

[B66] Pandey V. Srivastava A. Mishra M. Gaur R. K. (2022). Chilli leaf curl disease populations in India are highly recombinant, and rapidly segregated. Biotech 12, 1–12. 10.1007/s13205-022-03139-w 35251885 PMC8882514

[B67] Patil B. L. Fauquet C. M. (2010). Differential interaction between cassava mosaic geminiviruses and geminivirus satellites. J. Gen. Virol. 91, 1871–1882. 10.1099/vir.0.019513-0 20335493

[B68] Patil B. L. Ogwok E. Wagaba H. Mohammed I. U. Yadav J. S. Bagewadi B.. (2011). RNAi-mediated resistance to diverse isolates belonging to two virus species involved in Cassava brown streak disease. Mol. Plant Pathol. 12, 31–41. 10.1111/j.1364-3703.2010.00650.x 21118347 PMC6640250

[B69] Peele C. Jordan C. V. Muangsan N. Turnage M. Egelkrout E. Eagle P.. (2001). Silencing of a meristematic gene using geminivirus-derived vectors. Plant J. 27, 357–366. 10.1046/j.1365-313x.2001.01080.x 11532181

[B70] Perefarres F. Thierry M. Becker N. Lefeuvre P. Reynaud B. Delatte H.. (2012). Biological invasions of geminiviruses: case study of TYLCV and *Bemisia tabaci* in Reunion Island. Viruses 4, 3665–3688. 10.3390/v4123665 23235470 PMC3528285

[B71] Poornima Priyadarshini C. G. Ambika M. V. Tippeswamy R. Savithri H. S. (2011). Functional characterization of coat protein and V2 involved in cell to cell movement of Cotton leaf curl Kokhran virus-Dabawali. PLoS ONE 6:26929. 10.1371/journal.pone.0026929 22110597 PMC3217939

[B72] Powell P. A. Sanders P. R. Tumer N. Fraley R. T. Beachy R. N. (1990). Protection against tobacco mosaic virus infection in transgenic plants requires accumulation of coat protein rather than coat protein RNA sequences. Virology 175, 124–130 10.1016/0042-6822(90)90192-T 2309438

[B73] Rai V. P. Kumar R. Singh S. P. Kumar S. Kumar S. Singh M.. (2014). Monogenic recessive resistance to Pepper leaf curl virus in an interspecific cross of *Capsicum*. Sci. Horti. 172, 4–38. 10.1016/j.scienta.2014.03.039

[B74] Ramos R. S. Kumar L. Shabani F. Picanço M. C. (2019). Risk of spread of *Tomato yellow leaf curl virus* (TYLCV) in tomato crops under various climate change scenarios. Agric. Syst. 173, 524–535. 10.1016/j.agsy.2019.03.020

[B75] Ranjan K. Siddique R. A. Bharti M. K. Singh J. (2016). “Geminivirus: Indian scenario,” in Plant Viruses: Evolution and Management, eds R. K. Gaur, N. M. Nikolay Manchev Petrov, B. L. Patil and M. L. Stoyanova (Berlin: Springer), 131–144. 10.1007/978-981-10-1406-2_8

[B76] Roy A. Zhai Y. Ortiz J. Neff M. Mandal B. Mukherjee S. K.. (2019). Multiplexed editing of a *Begomovirus* genome restricts escape mutant formation and disease development. PLoS ONE 14:0223765. 10.1371/journal.pone.0223765 31644604 PMC6808502

[B77] Roy B. Dubey S. Ghosh A. Shukla S. M. Mandal B. Sinha P. (2021). Simulation of leaf curl disease dynamics in chili for strategic management options. Sci. Rep. 11, 1–12. 10.1038/s41598-020-79937-0 33441749 PMC7806845

[B78] Ruhel R. Chakraborty S. (2019). Multifunctional roles of geminivirus encoded replication initiator protein. Virus Dis. 30, 66–73. 10.1007/s13337-018-0458-0 31143833 PMC6517460

[B79] Sangeeta S. Tiwari A. K. (2017). “Begomoviruses: occurrence and management in Asia and Africa,” in Begomoviruses: Occurrence and Management in Asia and Africa, eds S. Saxena and A. Tiwari (Singapore: Springer), 2017.

[B80] Seal S. E. VandenBosch F. Jeger M. J. (2006). Factors influencing *Begomovirus* evolution and their increasing global significance: implications for sustainable control. Crit. Rev. Plant Sci. 25, 23–46. 10.1080/07352680500365257

[B81] Senanayake D. M. J. B. Jayasinghe J. E. A. R. M. Shilpi S. Wasala S. K. Mandal B. (2013). A new Begomovirus–betasatellite complex is associated with chili leaf curl disease in Sri Lanka. Virus Genes 46, 128–139. 10.1007/s11262-012-0836-4 23090833

[B82] Senanayake D. M. J. B. Mandal B. Lodha S. Varma A. (2007). First report of *Chilli leaf curl virus* affecting chili in India. Plant Pathol. 56:1513. 10.1111/j.1365-3059.2007.01513.x

[B83] Senanayake D. M. J. B. Varma A. Mandal B. (2012). Virus–vector relationships, host range, detection and sequence comparison of *Chilli leaf curl virus* associated with an epidemic of leaf curl disease of chilli in Jodhpur. Indian Phytopathol. 160, 146–155. 10.1111/j.1439-0434.2011.01876.x

[B84] Sharma P. Ikegami M. (2009). Characterization of signals that dictate nuclear/nucleolar and cytoplasmic shuttling of the capsid protein of Tomato leaf curl Java virus associated with DNAβ satellite. Virus Res. 144, 145–153. 10.1016/j.virusres.2009.04.019 19409945

[B85] Sharma V. K. Basu S. Chakraborty S. (2015). RNAi mediated broad-spectrum transgenic resistance in *Nicotiana benthamiana* to chili-infecting Begomoviruses. Plant Cell Rep. 34, 1389–1399. 10.1007/s00299-015-1795-8 25916177

[B86] Shih S. L. Tsai W. S. Green S. K. Khalid S. Ahmad I. Rezaian M. A.. (2003). Molecular characterization of tomato and chilli leaf curl Begomoviruses from Pakistan. Plant Dis. 87, 200–200. 10.1094/PDIS.2003.87.2.200A 30812928

[B87] Shih S. L. Tsai W. S. Green S. K. Singh D. (2007). First report of Tomato leaf curl Joydebpur virus infecting chilli in India. Plant Pathol. 56:341. 10.1111/j.1365-3059.2007.01540.x

[B88] Shukla A. K. Upadhyay S. K. Mishra M. Saurabh S. Singh R. Singh H.. (2016). Expression of an insecticidal fern protein in cotton protects against whitefly. Natl. Biotechnol. 34, 1046–1051. 10.1038/nbt.3665 27598229

[B89] Singh A. K. Kushwaha N. Chakraborty S. (2016). Synergistic interaction among begomoviruses leads to the suppression of host defense-related gene expression and breakdown of resistance in chilli. Appl. Microbiol. Biotechnol. 100, 4035–4049. 10.1007/s00253-015-7279-5 26780359

[B90] Singh O. W. Gupta D. Joshi B. Roy A. Mukherjee S. K. Mandal B. (2022). Spray application of a cocktail of dsRNAs reduces infection of chilli leaf curl virus in *Nicotiana benthamiana*. J. Plant Dis. Prot. 129, 433–438. 10.1007/s41348-021-00549-5

[B91] Soleymani S. Hakimitabar M. Seiedy M. (2016). Prey preference of predatory mite *Amblyseius swirskii* (Acari: Phytoseiidae) on *Tetranychus urticae* (Acari: Tetranychidae) and *Bemisia tabaci* (Hemiptera: Aleyrodidae). Biocont. Sci. Technol. 26, 562–569. 10.1080/09583157.2015.1133808

[B92] Srivastava A. Mangal M. Saritha R. K. Kalia P. (2017). Screening of chili pepper (*Capsicum spp*.) lines for resistance to the begomoviruses causing chili leaf curl disease in India. Crop Prot. 100, 177–185. 10.1016/j.cropro.2017.06.015

[B93] Tahir M. Haider M. S. Briddon R. W. (2010). Chilli leaf curl betasatellite is associated with a distinct recombinant begomovirus, Pepper leaf curl Lahore virus, in *Capsicum* in Pakistan. Virus Res. 149, 109–114. 10.1016/j.virusres.2009.12.007 20079779

[B94] Tashkandi M. Ali Z. Aljedaani F. Shami A. Mahfouz M. M. (2018). Engineering resistance against *Tomato yellow leaf curl virus via* the CRISPR/Cas9 system in tomato. Plant Signal Behav. 13:1525996. 10.1080/15592324.2018.1525996 30289378 PMC6204811

[B95] Thakur H. Jindal S. K. Sharma A. Dhaliwal M. S. (2018). *Chili leaf curl virus* disease: a serious threat for chilli cultivation. J. Plant Dis. Prot. 125, 239–249. 10.1007/s41348-018-0146-833100865

[B96] Thakur H. Jindal S. K. Sharma A. Dhaliwal M. S. (2019). A monogenic dominant resistance for leaf curl virus disease in chili pepper (*Capsicum annuum* L.). Crop Prot. 116, 115–120. 10.1016/j.cropro.2018.10.007

[B97] Thakur H. Jindal S. K. Sharma A. Dhaliwal M. S. (2020). Molecular mapping of dominant gene responsible for leaf curl virus resistance in chilli pepper (*Capsicum annuum* L.). 3 Biotech 10, 1–10. 10.1007/s13205-020-02402-2 32257738 PMC7103024

[B98] Thomas J. E. Gronenborn B. Harding R. M. Mandal B. Grigoras I. Randles J. W.. (2021). ICTV virus taxonomy profile: Nanoviridae. J. Gen. Virol. 2021:1544. 10.1099/jgv.0.001544 33433311 PMC8515864

[B99] Tian M. Wei Y. Zhang S. Liu T. (2017). Suitability of *Bemisia tabaci* (Hemiptera: *Aleyrodidae*) biotype-B and *Myzus persicae* (Hemiptera: *Aphididae*) as prey for the ladybird beetle, *Serangium japonicum* (Coleoptera: *Coccinellidae*). Eur. J. Entomol. 114, 603–608. 10.14411/eje.2017.073

[B100] Tsai W. S. Shih S. L. Green S. K. Rauf A. Hidayat S. H. Jan F. J. (2006). Molecular characterization of *Pepper yellow leaf curl Indonesia virus* in leaf curl and yellowing diseased tomato and pepper in Indonesia. Plant Dis. 90, 247–247. 10.1094/PD-90-0247B 30786428

[B101] Unseld S. Hohnle M. Ringel M. Frischmuth T. (2001). Subcellular targeting of the coat protein of African cassava mosaic geminivirus. Virology 286, 373–383. 10.1006/viro.2001.1003 11485405

[B102] Varma A. Malathi V. G. (2003). Emerging geminivirus problems: a serious threat to crop production. Ann. Appl. Biol. 142, 145–164. 10.1111/j.1744-7348.2003.tb00240.x

[B103] Vasudeva R. S. (1957). Report of the Division of Mycology and Plant Pathology. New Delhi: Indian Agricultural Research Institute.

[B104] Venkataravanappa V. Swarnalatha P. Reddy C. L. Chauhan N. Reddy M. K. (2016). Association of recombinant *Chili leaf curl virus* with enation leaf curl disease of tomato: a new host for chili Begomovirus in India. Phytoparasitica 44, 213–223. 10.1007/s12600-016-0510-9

[B105] Wang B. Yang X. Wang Y. Xie Y. Zhou X. (2018). *Tomato yellow leaf curl virus* V2 interacts with host histone deacetylase 6 to suppress methylation-mediated transcriptional gene silencing in plants. J. Virol. 92, e00036–e00018. 10.1128/JVI.00036-18 29950418 PMC6146709

[B106] Xie Y. Wu P. Liu P. Gong H. Zhou X. (2010). Characterization of alphasatellites associated with monopartite begomovirus/betasatellite complexes in Yunnan, China. Virol. J. 7, 178. 10.1186/1743-422X-7-178 20678232 PMC2922188

[B107] Yin K. Han T. Xie K. Zhao J. Song J. Liu Y. (2019). Engineer complete resistance to Cotton leaf curl Multan virus by the CRISPR/Cas9 system in *Nicotiana benthamiana*. Phytopathol. Res. 1, 1–9. 10.1186/s42483-019-0017-7

[B108] Zerbini F. M. Briddon R. W. Idris A. Martin D. P. Moriones E. Navas-Castillo J.. (2017). ICTV virus taxonomy profile: *Geminiviridae*. J. Gen. Virol. 98, 131–133. 10.1099/jgv.0.000738 28284245 PMC5802298

[B109] Zhou X. (2013). Advances in understanding begomovirus satellites. Ann. Rev. Phytopathol. 51, 357–381. 10.1146/annurev-phyto-082712-102234 23915133

