# Supplementary material for: An Overview of Chili Leaf Curl Disease: Molecular Mechanisms, Impact, Challenges, and Disease Management Strategies in Indian Subcontinent
Source: Front Microbiol. 2022 Jun 29;13:899512. doi: 10.3389/fmicb.2022.899512 (PMC9277185; doi:10.3389/fmicb.2022.899512)
Supplement: Supplementary file 1 [file Data_Sheet_1.docx]

**SUPPLEMENTARY TABLE 1│** Sources of resistance against ChiLCD were identified in different species of chili worldwide.

| **Sr. No** | **Genotype** | **Species** | **Country** | **Reference** |
| --- | --- | --- | --- | --- |
| 1 | BG-3821 | *C. chinense* | Mexico | Adluri et al., 2017; Mejia-Teniente, 2015 |
| 2 | Bhut Jolokia | *C. chinense* | Mexico, India | Adluri et al., 2017; Rai et al., 2014 |
| 3 | BS-35, GKC-29 and EC-497636 | *C. annuum* | India | Srivastava et al., 2017 |
| 4 | PBC 143, PBC 144, PBC 149, PBC 495, and VI012005 | *C. annuum* | Taiwan | Kenyon et al., 2014 |
| 5 | BS-35, and GKC-29 | *C. annuum* | India | Bhutia et al., 2015 |
| 6 | ‘Kalyanpur Chanchal’ | *C. annuum* | India | Singh et al., 2016 |
| 7 | DLS-Sel-10,  WBC-Sel-5, PBC-142 and PBC-535 | *C. annuum* | India | Dhaliwal et al., 2015 |
| 8 | S-343 | *C. annuum* | India | Thakur et al., 2020 |
| 9 | DLS-Sel-10 | *C. annuum* | India | Thakur et al., 2019 |
| 10 | Punjab Lal | *C. annuum* | India | Kushwaha et al., 2015 |

**SUPPLEMENTARY FIGURE 1│** Geographical distribution of *Chili leaf curl virus* (ChiLCV) infecting chili worldwide (Data obtained from [https://batchgeo.com/map/1707bc1a1d 04711679027a 2225c293e5](https://batchgeo.com/map/1707bc1a1d%2004711679027a%202225c293e5)). The red dots indicated in this figure are the states or countries denoted for the presense of ChiLCV in chili worldwide.
